# Exploring the Mechanisms of Traditional Chinese Herbal Therapy in Gastric Cancer: A Comprehensive Network Pharmacology Study of the Tiao-Yuan-Tong-Wei decoction

**DOI:** 10.3390/ph17040414

**Published:** 2024-03-25

**Authors:** Juan Chen, Jingdong Kang, Shouli Yuan, Peter O’Connell, Zizhu Zhang, Lina Wang, Junying Liu, Rongfeng Chen

**Affiliations:** 1 Department of Gastroenterology, Beijing Nuclear Industry Hospital, Beijing 102413, China; chenjuan672464@163.com (J.C.);; 2 Department of General Surgery, Beijing Nuclear Industry Hospital, Beijing 102413, China; 3 Academy for Advanced Interdisciplinary Studies, Peking University, Beijing 100871, China; yuanshouli123@163.com; 4 School of Pharmacy and Pharmaceutical Sciences, Trinity College Dublin, D02 PN40 Dublin, Ireland; 5 Pharmacy Department, Beijing Water Resources Hospital, Beijing 100036, China; 6 National Center for Occupational Safety and Health, National Health Commission, Beijing 102308, China

**Keywords:** gastric cancer, Tiao-Yuan-Tong-Wei decoction, agrimoniin, baicalin, corosolic acid, CASP3, PIK3CA

## Abstract

The use of herbal medicine as an adjuvant therapy in the management of gastric cancer has yielded encouraging outcomes, notably in enhancing overall survival rates and extending periods of disease remission. Additionally, herbal medicines have demonstrated potential anti-metastatic effects in gastric cancer. Despite these promising findings, there remains a significant gap in our understanding regarding the precise pharmacological mechanisms, the identification of specific herbal compounds, and their safety and efficacy profiles in the context of gastric cancer therapy. In addressing this knowledge deficit, the present study proposes a comprehensive exploratory analysis of the Tiao-Yuan-Tong-Wei decoction (TYTW), utilizing an integrative approach combining system pharmacology and molecular docking techniques. This investigation aims to elucidate the pharmacological actions of TYTW in gastric pathologies. It is hypothesized that the therapeutic efficacy of TYTW in counteracting gastric diseases stems from its ability to modulate key signaling pathways, thereby influencing PIK3CA activity and exerting anti-inflammatory effects. This modulation is observed predominantly in pathways such as PI3K/AKT, MAPK, and those directly associated with gastric cancer. Furthermore, the study explores how TYTW’s metabolites (agrimoniin, baicalin, corosolic acid, and luteolin) interact with molecular targets like AKT1, CASP3, ESR1, IL6, PIK3CA, and PTGS2, and their subsequent impact on these critical pathways and biological processes. Therefore, this study represents preliminary research on the anticancer molecular mechanism of TYTW by performing network pharmacology and providing theoretical evidence for further experimental investigations.

## 1. Introduction

Gastric cancer is a significant health concern worldwide, with China accounting for approximately half of all cases [1]. Traditional Chinese medicine (TCM) has been used for centuries to treat various diseases, including gastric cancer. In recent years, there has been growing interest in comparing the effectiveness of TCM with modern treatments for gastric cancer [2]. Traditional Chinese formulas have been used to treat gastric cancer, although the specific formulas may vary. The use of herbal remedies and complementary and alternative medicine, including traditional Chinese medicine, is common among cancer patients [3]. Further research is needed to explore the specific traditional Chinese formulas used and their effectiveness in treating gastric cancer. Therefore, the potential of herbal medicine as a therapy for gastric cancer has not been explored thoroughly, and the molecular targets of herbal medicine and the potential therapeutic mechanisms remain poorly understood. In TCM, decoctions like Tiao-Yuan-Tong-Wei (TYTW) are typically created by boiling a mixture of selected herbs. These herbs are chosen based on TCM principles that focus on restoring and maintaining balance within the body. Each herb in a decoction is believed to contribute its unique properties, and when combined, they are thought to work synergistically to enhance the overall therapeutic effect. The name “Tiao Yuan Tong Wei” gives some insight into the intended purpose of the decoction within the TCM framework: “Tiao” often implies regulation or adjustment, suggesting that the decoction aims to regulate some aspect of bodily function. “Yuan” can refer to the source or origin, which in TCM might relate to the fundamental life energies or core bodily systems. “Tong-Wei” implies a focus on the stomach and digestion, as “Tong” often means to unblock or pass through, and “Wei” is related to the stomach or digestion in TCM.

The most common gastric cancer symptoms are gastrointestinal, including dry mouth, weight loss, early satiety, taste change, constipation, anorexia, bloating, nausea, abdominal pain, and vomiting [4]. Other symptoms include indigestion, heartburn, diarrhea or constipation, blood in the stool, and weight loss [5]. Symptoms are often nonspecific and may be indistinguishable from those of benign dyspepsia, while the presence of alarm symptoms may imply an advanced and often inoperable disease [6]. These studies suggest that several factors increase the risk of developing gastric cancer. Ramos (2018) and his colleague identified symptoms of *Helicobacter pylori*, low fruit and vegetable intake, high salt intake, smoking, and alcohol consumption as risk factors [7]. Yusefi et al. (2018) identified 52 risk factors: diet, lifestyle, genetic predisposition, family history, treatment, medical conditions, infections, demographic characteristics, occupational exposures, and ionizing radiation [8]. Martel et al. (2013) also highlighted infection with *H. pylori* as the most important factor in non-cardia gastric cancer [9].

The survival rates for gastric cancer vary depending on the study and the population being studied. Iran’s 5-year survival rate was 12.8% [10]. A meta-analysis of 21 randomized studies found a significant survival benefit for patients who received adjuvant systemic chemotherapy postoperatively [11]. The overall five-year survival rate in Korea was 66.8%, and researchers identified several clinicopathologic factors significantly associated with survival rates [12]. The median survival time in Iran was 24.2 months, and several independent prognostic factors were identified, including tumor stage, histology grade, histologic type of cancer, tumor size, age at diagnosis, and surgical approach [13]. Overall, these papers suggest that survival rates for gastric cancer are generally low but can be improved with early detection, appropriate surgery, and adjuvant therapy. The treatment landscape for gastric cancer has evolved in recent years. Systemic chemotherapy is still the mainstay treatment for metastatic disease. Still, the introduction of agents targeting human epidermal growth factor receptor two and vascular endothelial growth factor/vascular endothelial growth factor receptor has brought this disease into the era of molecular and personalized medicine [14]. Surgery remains the only curative therapy, while perioperative and adjuvant chemotherapy and chemoradiation can improve the outcome of resettable gastric cancer with extended lymph node dissection. Cisplatin and fluoropyrimidine-based chemotherapy, with the addition of trastuzumab in human epidermal growth factor receptor two-positive patients, is a widely used treatment in stage IV patients fit for chemotherapy [15].

A meta-analysis evaluated the efficacy of TCM combined with chemotherapy in treating gastric cancer [16]. The results showed that the 3-year survival rate of gastric cancer patients in the TCM combination group was higher than that in the chemotherapy alone group (OR = 1.71, 95% CI 1.06–2.78, Z = 2.18, *p* = 0.03). There was no heterogeneity among studies (χ^2^ = 2.18, *p* = 0.54, I^2^ = 0%), and there was no significant publication bias (*p* > 0.05). The incidence of nausea and vomiting after chemotherapy in gastric cancer patients in the TCM combination group was lower than in the chemotherapy alone group (OR = 0.47, 95% CI 0.34–0.64, Z = 4) [16]. However, it is essential to note that the study is the only reference provided that directly compares traditional Chinese formulas to modern treatments for gastric cancer. Therefore, based solely on this reference, it is not easy to draw definitive conclusions about the overall effectiveness of TCM compared with current therapies. In contrast, the study found that Chinese gastric cancer patients had a worse outcome than U.S. gastric cancer patients, even after adjusting for important prognostic factors. The differences in survival between the two countries may be attributed to factors such as patient characteristics, tumor characteristics, and treatment approaches. Furthermore, the study highlighted differences in gastric cancer characteristics between China and other Asian countries. For example, Chinese patients had larger tumors and a later presentation stage than Japanese or Korean patients [1]. Proximal gastric cancer was also higher in China than in Japan and Korea [1]. It is important to note that traditional Chinese formulas for treating gastric cancer may vary depending on individual preferences, cultural practices, and the advice of traditional Chinese medicine practitioners. The specific procedures used may also depend on the stage and severity of the cancer and the overall treatment plan. This study included the first comprehensive exploratory analysis of the Tiao-Yuan-Tong-Wei decoction (TYTW)’s pharmacological mechanism of action (MOA) on gastric cancer, using the potent combination of system pharmacology and molecular docking. This approach provides a new, lean strategy to focus the development of herb-based therapeutics.

## 2. Results

### 2.1. Compound Identification of TYTW and the ADMET Prediction

As a result, 100 ingredients were found in the Traditional Chinese Medicine Systems Pharmacology Database and Analysis Platform (TCMSP) and the Encyclopaedia of Traditional Chinese Medicine (ETCM) by searching keywords of nine herb species. Only the major component was collected according to the literature validation listed in Table 1. Other compounds were obtained by searching the Bioinformatics Analysis Tool for Molecular Mechanism of TCM (BATMAN-TCM) (score > 10, *p* < 0.05), Traditional Chinese Medicine Integrated Database (TCMID), and SymMap (OB > 30). *Radix pseudostellariae* consists of six major active metabolites (1-monolinolein, mcacetin, beta-sitosterol, luteolin, schottenol, taraxerol), *Ophiopogonis japonicus* is composed of four major active metabolites (ruscogenin, ophiopogonanone B, linalool, and adenosine), *Scutellaria baicalensis* consists of tetramethoxyflavone, scutevulin, dimethoxyflavone, baicalin, baicalein, and 7-methoxybaicalein, *Agrimoniae herba* consists of agrimoniin, catechin, dihydroquercetin, quercetin, *Citrus aurantium* consists of prunasin, neohesperidin, naringin, hesperetin, ephedrine, *Salvia miltiorrhiza* consists of cryptotanshinone, danshenol A, danshenol B, danshensu, isocryptotanshinone, isotanshinone I, salviol, tanshinol A, and tanshinone I; *Actinidia valvata* consists of asiatic acid, aorosolic acid, and netursen-28-oic acid, *Radix codonopsis lanceolatae* of cycloartenol, echinocystic acid, and oleanolic acid, *Radix glycyrrhizae preparata* of glycyrrhizic acid, isoliquiritin, liquiritigenin, and liquiritin. The components with a good drug-likeness grading were acacetin, ophiopogonanone B, wogonin, tetramethoxyflavone, scutevulin, dimethoxyflavone, baicalein, 7-methoxybaicalein, hesperetin, hphedrine, cryptotanshinone, danshenol A, danshenol B, isocryptotanshinone, salviol, tanshinol A, and liquiritigenin (Table 1).

### 2.2. Drug Target and Disease Target Prediction

**Figure 1:**
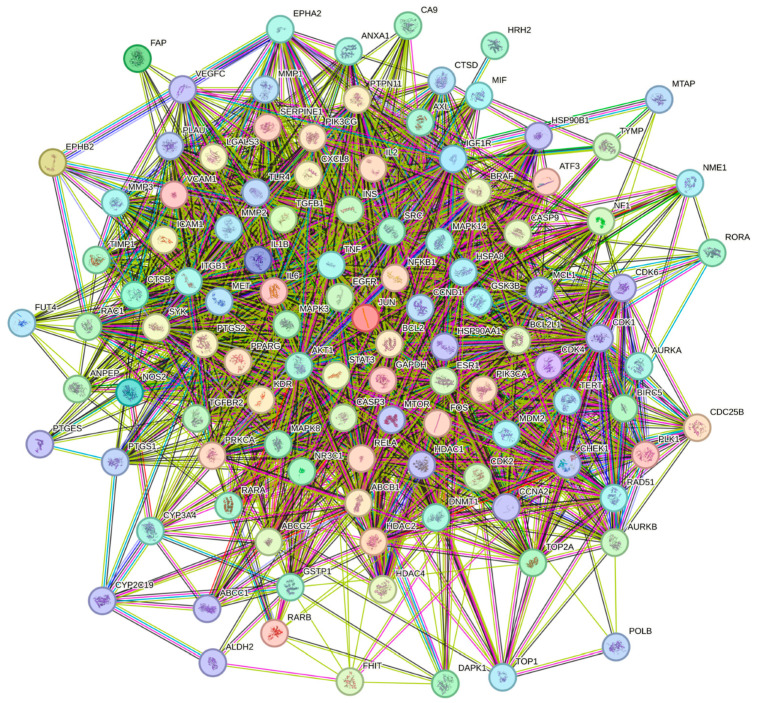
PPI network of total overlapping targets. There is no particular meaning of the node color itself.

Twenty-three components were obtained by searching ETCM with the screening criterion of drug-likeness (DL) grading equal to moderate or good. The targets of each bioactive compound from the ETCM database were selected based on a screening score of >0.8, resulting in 165 genes related to TYTW’s compounds. Subsequently, targets were not predicted in the ETCM database search for several compounds purported as bioactive components of TYTW in the relevant literature. Instead, three public web servers, TargetNet, SwissTargetPrediction, and PharmMapper, were used to predict the potential targets of these bioactive components (agrimoniin, prunasin, tanshinone I, netursen-28-oic acid, and corosolic acid) according to the similarity-based method. The targets obtained from SwissTargetPrediction and PharmMapper were 158, 56, 11, 81, and 89, respectively. Therefore, 45 components were retained following the database and literature searches. Human disease targets from Genecards, MalaCards, and DisGeNET were 396, 58, and 189, respectively. Finally, the drug targets obtained for each component of TYTW were intersected with genes associated with gastric cancer, as shown in the Venn diagram of the intersected gene symbols. The overlapping target was imported in STRING to obtain the protein–protein interaction (PPI) network, which visualizes the connections of each gene (Figure 1).

### 2.3. Herb–Compounds–Targets–Disease Network of TYTW Analysis

**Figure 2:**
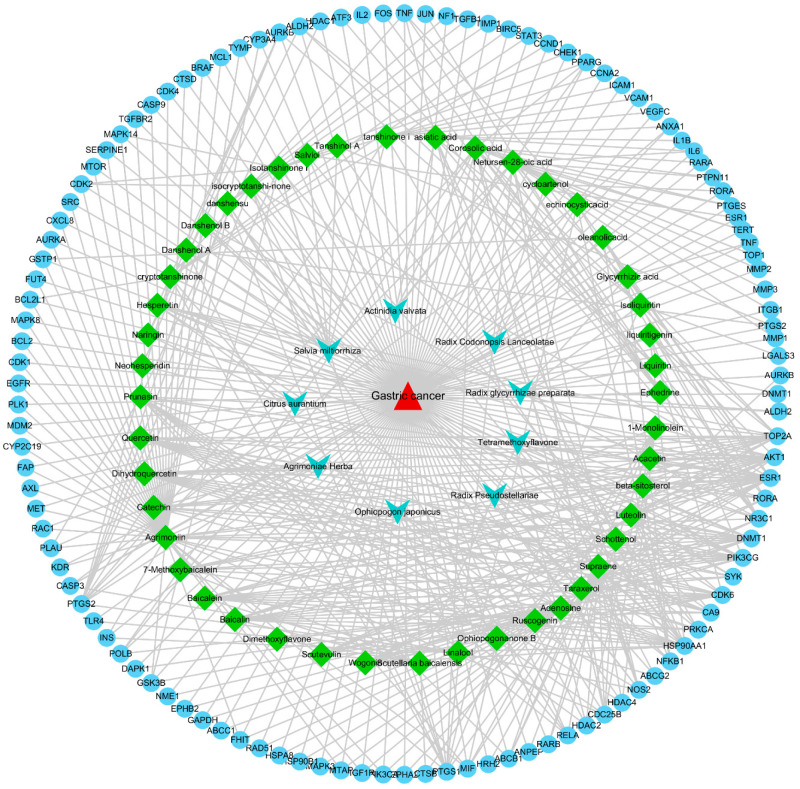
Disease–targets–component–herb network. The red triangle node represents the formula of TYTW, and the azure arrow node represents herb species. The green diamonds represent the bioactive compounds, and the blue circles represent the overlapping targets between herbs and disease.

Cytoscape was used to visualize and analyze the herb–compounds–targets–disease (HCTD) network, elucidating how the herb may act in gastric cancer. This produced a network of complex information based on the interactions between the formula (TYTW), compounds, gene symbols (targets), and disease (gastric cancer). The red triangle node represents the formula of TYTW, and the azure arrow node represents herb species. The green diamonds represent the bioactive compounds, and the blue circles represent the overlapping targets between herbs and disease. The network comprised 117 targets assigned to 46 compounds, indicating HCTD interactions (Figure 2). Finally, the Cytoscape plugin, a network analyzer, was used to determine the hub nodes where the degree and closeness of the node satisfied specific criteria, such as the median of the corresponding parameters. The hub gene symbol screening criteria based on closeness centrality > 0.5 and degree > 10 resulted in 10 core gene symbols. Based on the screening criteria, the top four compounds were selected for validation by molecular docking, including agrimoniin, baicalin, corosolic acid, and luteolin, and top targets included protein kinase B (AKT1), apoptosis-related cysteine peptidase (CASP3), estrogen receptor 1 (ESR1), interleukin 6 (IL6), phosphatidylinositol 3-kinase catalytic subunit alpha (PIK3CA), and prostaglandin–endoperoxide synthase (PTGS2).

### 2.4. The Gene Ontology (GO) and Kyoto Encyclopedia of Genes and Genomes (KEGG) Enrichment Analysis

**Figure 3:**
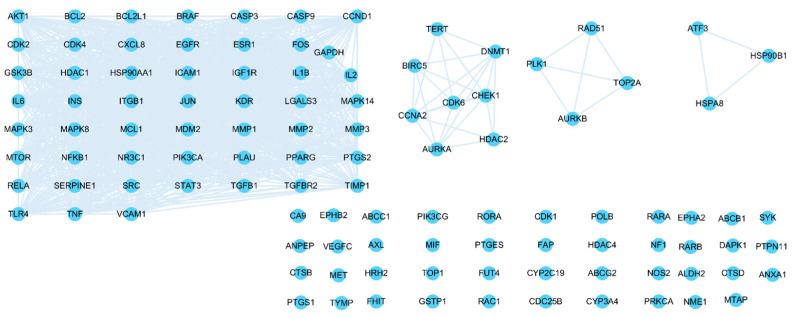
Minimal Common Oncology Data Elements (MCODE) clustering (four clusters).

**Figure 4:**
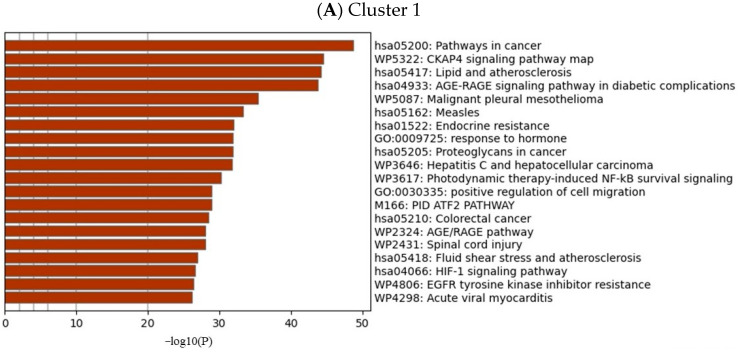

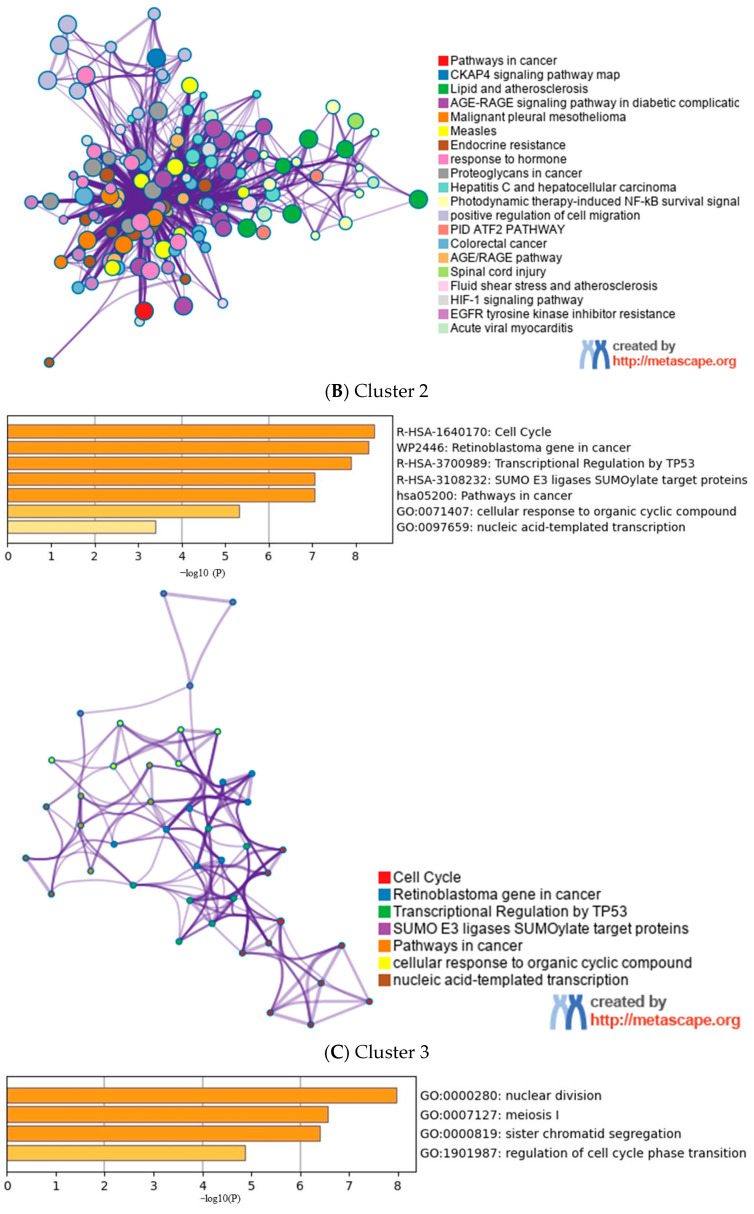

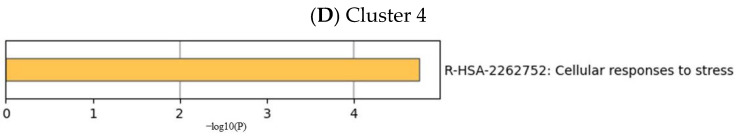
GO and KEGG enrichment of each cluster following MCODE clustering in Cytoscape.

**Figure 5:**
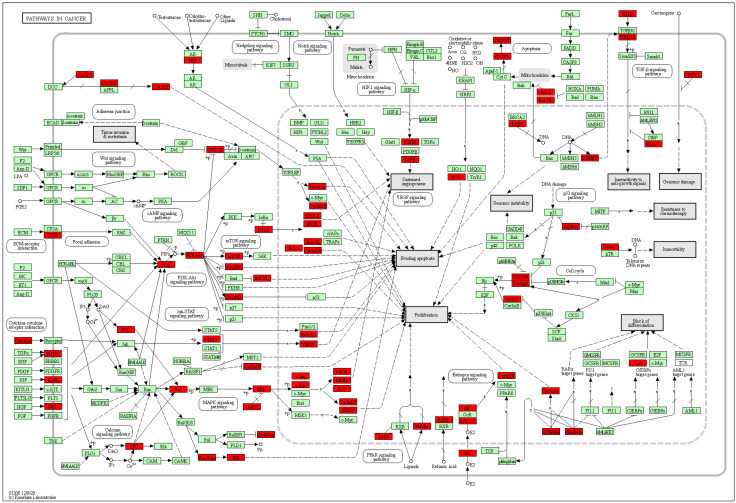
KEGG pathway (hsa05200 pathway in cancer). The red nodes represent up-regulated genes while the green ones are down-regulated genes. Two genes are linked together with solid lines if they function together in the present KEGG pathways while those connected with dotted lines do not function together.

**Figure 6:**
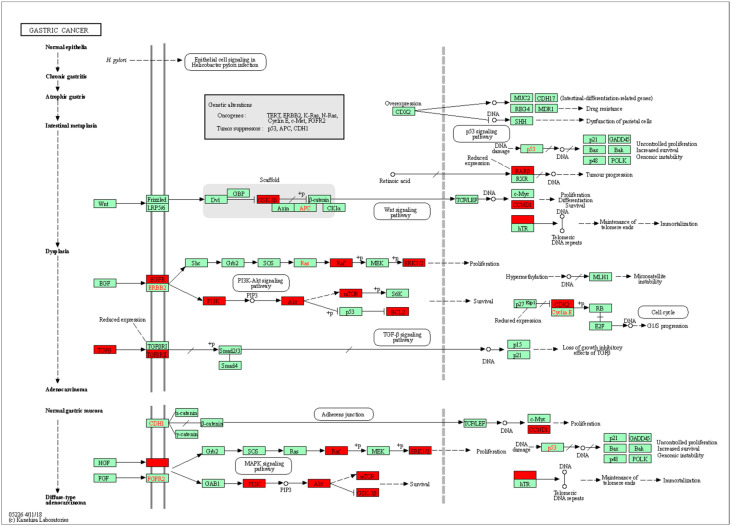
KEGG pathway (hsa05226 gastric cancer). The red nodes represent up-regulated genes while the green ones are down-regulated genes. Two genes are linked together with solid lines if they function together in the present KEGG pathways while those connected with dotted lines do not function together.

**Figure 7:**
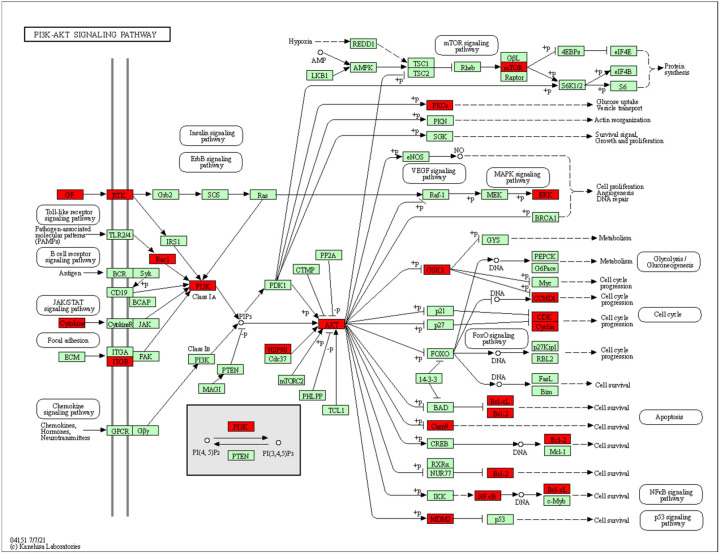
KEGG pathway (hsa04151 P13K-AKT signaling pathway). The red nodes represent up-regulated genes while the green ones are down-regulated genes. Two genes are linked together with solid lines if they function together in the present KEGG pathways while those connected with dotted lines do not function together.

The intersected genes were imported to Cytoscape and analyzed by the MCODE clustering algorithm, identifying functional subnetworks within Cytoscape. MCODE clustering analysis identified four clusters (Figure 3). As shown in Table 2 and Figure 4, 65 key targets and 19 key pathways were identified from the four clusters, thus representing potential core targets for TYTW in treating gastric cancer. Cluster 1 includes pathways such as pathways in cancer (Figure 5), cytoskeleton-associated protein 4 (CKAP4) signaling pathway map, lipid and atherosclerosis, malignant pleural mesothelioma, gastric cancer (Figure 6), PI3K-Akt signaling pathway (Figure 7), endocrine resistance, response to hormone, proteoglycans in cancer, hepatitis C and hepatocellular carcinoma, photodynamic therapy-induced NF-kB survival signaling. The GO and KEGG enrichment in the Database for Annotation, Visualisation and Integrated Discovery (DAVID) showed the TYTW targeted at stomach neoplasms, malignant neoplasm of the stomach, and hereditary diffuse gastric cancer; GO term enrichment includes protein phosphorylation, protein serine/threonine/tyrosine kinase activity, ATP binding, protein serine/threonine kinase activity, peptidyl-serine phosphorylation, protein kinase activity, and kinase activity (Table 3).

### 2.5. Molecular Validation of Compound-Target Interactions

**Figure 8:**
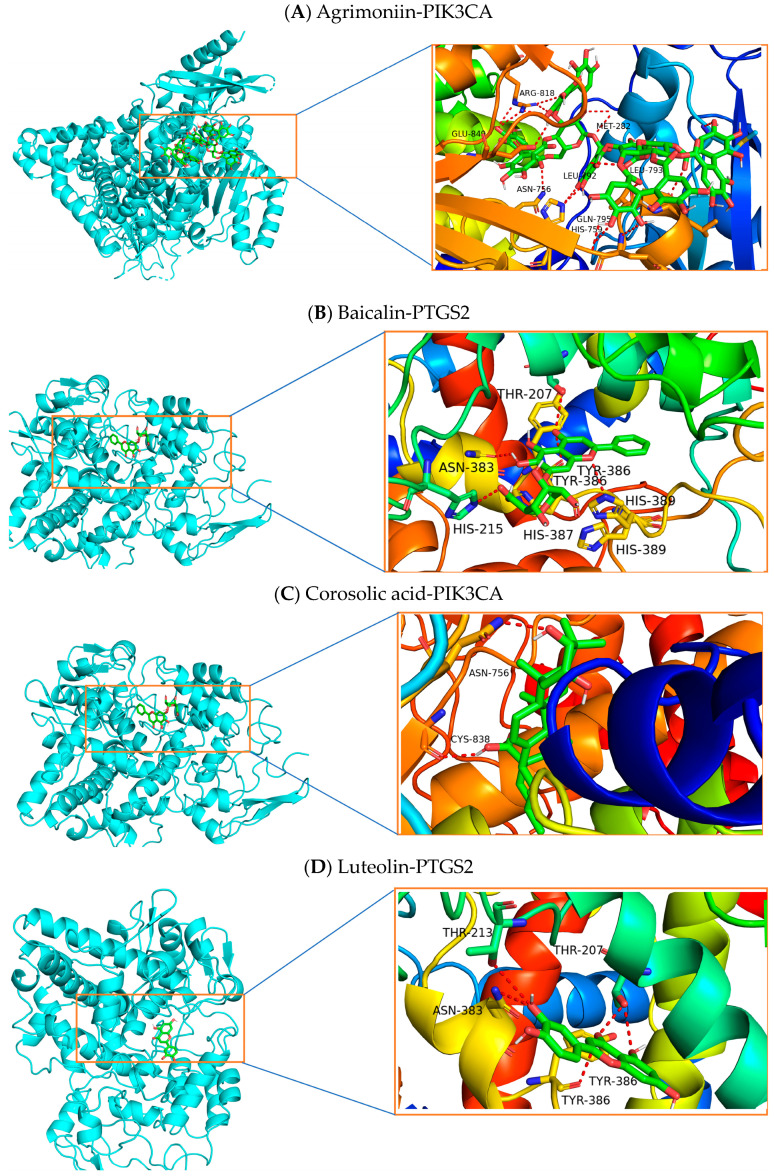
Molecular docking of the complex with the best binding affinity of each component. The binding affinity is shown in Table 4. In the left of binding complex, the cyan color represents protein while green one represents small molecule. In the right of binding pocket, there is no meaning for the color itself.

Based on the results of the KEGG pathway analysis and PPI network analysis, the critical related targets were selected for molecular docking validation. The crystal structures of key target proteins were obtained from RCSB PDB, including AKT1, CASP3, ESR1, IL6, PIK3CA, and PTGS2 and the component structure was obtained from PubChem, including agrimoniin, baicalin, corosolic acid, and luteolin (Table 4). Molecular docking representations for agrimoniin-AKT1 (−11.3 kcal/mol), agrimoniin-PIK3CA (−12.0 kcal/mol), agrimoniin-PTGS2 (−9.6 kcal/mol), baicalin-PIK3CA (−9.0 kcal/mol), baicalin-PTGS2 (−10.0 kcal/mol), corosolic acid-PIK3CA (−9.9 kcal/mol), and luteolin-PIK3CA (−9.3 kcal/mol) are shown in Figure 8. Binding affinities (kcal/mol) in each case were <−8 kcal/mol, indicating strong binding. Agrimoniin demonstrated the best pose in the PIK3CA catalytic site ribbon model (agrimoniin-PIK3CA complex, Figure 8A) and performed interactions with amino acid residues in the PIK3CA active site, such as TET-282, ARG-852, ARG-818, and HIS-759.

**Figure 9:**
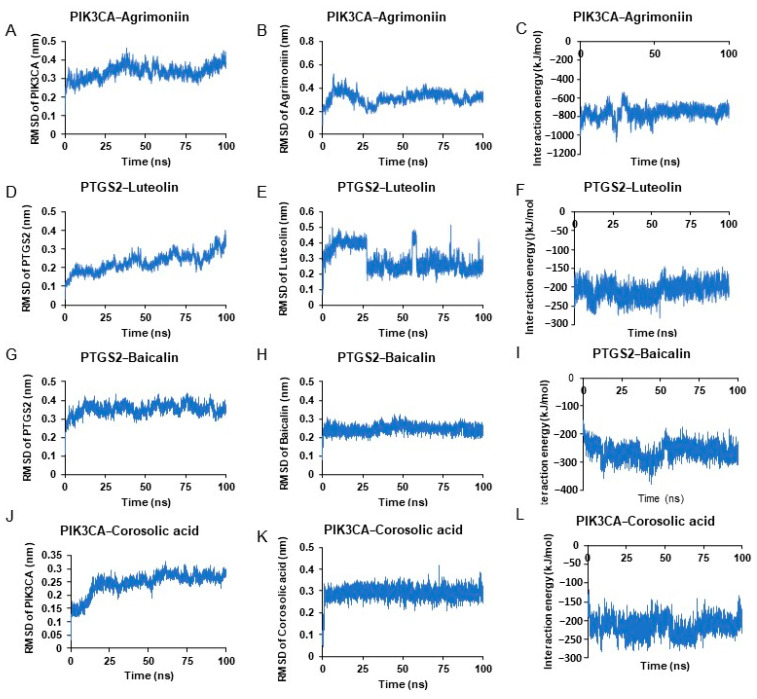
Molecular dynamics simulation results selected based on the molecular docking in Figure 8. RMSD of docked complexes has been run at 100 ns. (**A**–**C**) represent the PIK3CA-Agrimoniin complex; (**D**–**F**) for PTGS2-Luteolin complex; (**G**–**I**) for PTGS2-Baicalin complex; and (**J**–**L**) for PIK3CA-Corosolic acid complex.

In order to verify the stability of the docking structures, we selected agrimoniin-PIK3CA, corosolic acid-PIK3CA, baicalin-PTGS2, and luteolin-PIK3CA complexes for dynamic simulation analysis. As shown in Figure 9, the RMSD of proteins and small molecules in the complex structures remained relatively stable during the simulation, especially the baicalin-PTGS2 and corosolic acid-PIK3CA complexes. The RMSD of luteolin in luteolin-PIK3CA varied greatly, and it was stable at 0.3 nm compared with the initial docking structure, indicating that the position of luteolin changed significantly during the simulation process. This position change occurred rapidly and the luteolin remained stable at the new binding position. The average interaction energy of agrimoniin-PIK3CA, corosolic acid-PIK3CA, baicalin-PTGS2, and luteolin-PIK3CA complexes was −802.34 kJ/mol, −176.39 kJ/mol, −183.82 kJ/mol, and −216.77 kJ/mol.

## 3. Discussion

While some evidence suggests that TCM combined with chemotherapy may positively impact the outcomes of gastric cancer patients, more research is needed to fully understand the effectiveness of traditional Chinese formulas compared with modern treatments. The study supports the use of TCM in gastric cancer treatment, but additional studies are required to validate these findings. It is also important to consider the differences in patient characteristics, tumor characteristics, and treatment approaches between countries when comparing gastric cancer outcomes.

TYTW consists of nine herb species. *R pseudostellariae*, also known as *Pseudostellaria heterophylla*, is a medicinal plant widely used in Chinese clinical practice for its hypoglycemic effect. It contains polysaccharides, which have been found to have a hypoglycemic effect in treating diabetes mellitus [17]. In addition to its anti-diabetic properties, *R. pseudostellariae* has also been found to have other biological activities. It contains volatile compounds, saponins, cycle peptides, amino acids, and minerals related to diverse biological activities, such as suppressing cough and enhancing the immune system [18]. These properties make *R. pseudostellariae* a valuable herb in Chinese medicine. *O. japonicus*, also known as *Ophiopogonis radix*, is a TCM that has been investigated for its therapeutic properties in cardiovascular diseases [19]. Studies have shown that *O. japonicus* has effects on cardiovascular diseases through various mechanisms, including antioxidation, antiarrhythmia, and microcirculation [20]. Therefore, the anti-inflammatory properties of *O. japonicus* may contribute to its potential role in cancer treatment. Results showed that the extract ameliorated oxidative stress and inflammatory response, indicating its potential to protect the heart from damage caused by doxorubicin, a chemotherapy drug commonly used in cancer treatment [20]. Furthermore, another study investigated the anti-inflammatory activity of ruscogenin, a compound found in *O. japonicus*. The study found that ruscogenin inhibited the expression of intercellular adhesion molecule-1 (ICAM-1) and nuclear factor-κB (NF-κB), both of which are involved in the inflammatory response [21]. This suggests that *O. japonicus* may have anti-inflammatory effects that could benefit cancer treatment.

*S. baicalensis* has many applications in traditional medicine, including treating diarrhea, dysentery, hypertension, hemorrhaging, insomnia, inflammation, and respiratory infections. The major bioactive compounds extracted from the root of *S. baicalensis* are flavones, such as baicalin, wogonoside, baicalein, and wogonin [22]. The flavones extracted from *S. baicalensis* have been found to possess various pharmacological activities, including antioxidant, anti-inflammatory, and anticancer properties. In several studies, baicalin, one of the major flavones in *S. baicalensis*, has been shown to inhibit the growth and proliferation of cancer cells. Baicalin has been found to induce apoptosis, or programmed cell death, in cancer cells through various mechanisms [23]. It can activate caspases, which are enzymes involved in the apoptotic process, and inhibit the expression of anti-apoptotic proteins. Baicalin has also been shown to inhibit the migration and invasion of cancer cells, which are important processes in cancer metastasis. In addition to baicalin, other flavones from *S. baicalensis*, such as baicalein and wogonin, have also demonstrated anticancer effects. Baicalein has been found to inhibit the growth of various cancer cells, including breast, lung, and prostate cancer cells [24]. Wogonin has shown potential in suppressing proliferation and inducing apoptosis in cancer cells [25].

Furthermore, studies have indicated that consumption of the roots and shoots of *S. baicalensis* can inhibit mutagenesis, a process of genetic mutation that can lead to cancer development [26]. This suggests that *S. baicalensis* may have a protective effect against the initiation and progression of cancer. Therefore, *S. baicalensis* has shown potential in cancer treatment, particularly its flavones, such as baicalin, baicalein, and wogonin. These compounds have demonstrated anticancer effects by inducing apoptosis, inhibiting cell growth and proliferation, and suppressing migration and invasion of cancer cells. Additionally, *S. baicalensis* has been found to inhibit mutagenesis, suggesting a potential protective effect against cancer development. Further research is needed to fully understand the mechanisms and possible applications of *S. baicalensis* in cancer treatment.

*A. herba*, known as agrimony, has been studied for its potential anticancer properties and its traditional use in folk medicine. *A. herba* contains various bioactive compounds, including flavonoids, tannins, and phenolic acids, associated with potential anticancer effects. These compounds possess antioxidant and anti-inflammatory properties, which are important in cancer prevention and treatment [27]. A study investigating the anticancer activity of *A. eupatoria* extract showed that it inhibited the growth of gastric cancer cells in vitro. The extract induced cell cycle arrest and apoptosis in the cancer cells, suggesting its potential as a therapeutic agent [28]. Furthermore, another study evaluated the chemopreventive effects of *A. eupatoria* extract in a rat model of gastric cancer. The extract was found to reduce the incidence and multiplicity of gastric tumors, indicating its potential in preventing the development of gastric cancer [28].

Based on our analysis, the major targets of TYTW are AKT1, CASP3, ESR1, IL6, PIK3CA, and PTGS2. Research has discussed the role of the Akt-p53-miR-365-cyclin D1/cdc25A axis in gastric tumorigenesis induced by PTEN deficiency; the study investigated the molecular mechanisms underlying gastric tumorigenesis in the context of PTEN deficiency [29]. PTEN is a tumor suppressor gene that regulates cell growth and survival by inhibiting the Akt signaling pathway. Loss of PTEN function is commonly observed in various cancers, including gastric cancer. The researchers found that loss of PTEN in gastric epithelial cells resulted in gastric cancer within the first two months of age. Concurrent ablation of Akt1 rescued the tumor growth induced by PTEN deficiency. The study demonstrated a cell-autonomous function of epithelial PTEN in suppressing gastric tumor formation and the role of intrinsic Akt overactivation in promoting gastric cell proliferation. Overall, the study highlights the importance of the Akt signaling pathway and its downstream targets in gastric tumorigenesis. The dysregulation of this pathway, particularly in the context of PTEN deficiency, can promote cell proliferation and survival, leading to the development of gastric cancer.

CASP3 plays a complex role in gastric cancer. Its downregulation may contribute to altered chemoresistance and the escape of cancer cells from apoptosis [30]. Additionally, CASP3 has been associated with tumor invasion, metastasis, and angiogenesis. CASP3 has been implicated in several aspects of the disease. Studies have shown that alterations in CASP3 expression and activity can contribute to tumorigenesis and cancer progression. Downregulation of CASP3 has been observed in many human cancers, including gastric cancer [31]. Reduced CASP3 expression may be associated with altered chemoresistance and the escape of cancer cells from apoptosis [31]. Furthermore, CASP3 involves pathways associated with controlling neuronal cytoskeletal components and their regulators, such as actin, microtubule-associated protein 2 (MAP2), growth-associated protein 43 (GAP43), drebrin 1 (DBN1), and calmodulin [32]. This suggests that CASP3 may play a role in fine-tuning intercellular connections and tissue regeneration. In addition, CASP3 has been associated with tumor invasion, metastasis, and angiogenesis in various cancers [33]. Studies have shown that CASP3 promotes tumor growth by creating a microenvironment that supports angiogenesis [33]. In colon cancer, CASP3 has been implicated in tumor invasion and metastasis, and its deletion often indicates higher sensitivity to chemotherapy and radiation [33].

ESR1 encodes estrogen receptor α (ERα), which can interact with other signaling molecules through multiple pathways, such as the PI3K/AKT or mitogen-activated protein kinase (MAPK) signaling pathway [34]. The ESR1 mutation has been associated with cell proliferation, metastasis, and invasion in gastric cancer [35]. PIK3CA mutations have been observed in various cancer types, including breast and hepatocellular carcinoma. PIK3CA mutations have been detected in gastric cancer, albeit at a lower frequency than breast and hepatocellular carcinomas [36]. The incidence of PIK3CA mutations in gastric cancer has been reported to be around 6.5% [37]. Some of these mutations were detected in early lesions of gastric cancer, suggesting that PIK3CA mutation may occur independently of the stage of the tumors [38]. Overexpression of the PIK3CA gene in gastric cancer has been suggested as an indication for targeted therapeutic drugs such as sorafenib [39]. Additionally, when the PIK3CA gene is found to have a mutation, 5-Aza has been recommended as a target therapeutic drug. Therefore, PIK3CA mutations have been observed in gastric cancer, albeit at a lower frequency than other cancer types. The PIK3CA gene has been identified as a promising biomarker in gastric cancer, and its overexpression or mutation status may guide targeted therapeutic approaches. Further research is needed to fully understand the role of PIK3CA in gastric cancer and its potential implications for diagnosis and treatment. PTGS2 is upregulated in gastric cancer and may play a role in tumor progression. PTGS2 is an enzyme involved in the synthesis of prostaglandins, which are lipid mediators involved in various physiological and pathological processes, including inflammation and cancer [40]. In gastric cancer, PTGS2 is upregulated and associated with tumor progression and poor prognosis. Studies have shown that PTGS2 expression increases in gastric cancer cells treated with tanshinone IIA (Tan IIA), a compound derived from traditional Chinese medicine [41]. The upregulation of PTGS2 was observed in response to Tan IIA treatment, along with increased lipid peroxidation [42]. PTGS2 has been extensively studied and is associated with inflammation and carcinogenesis. PTGS2 mRNA levels were significantly higher in intestinal tissues from colorectal adenoma and cancer cases than healthy controls [43]. However, the role of PTGS2 in gastric cancer may differ from its role in colorectal cancer. Further research is needed to fully understand the mechanisms and functional implications of PTGS2 in gastric cancer and its potential as a therapeutic target.

TYTW targets several pathways. Firstly, the PI3K-Akt signaling pathway plays a significant role in gastric cancer. Activation of this pathway has been associated with tumor growth, survival, invasion, and metastasis in gastric cancer cells [44]. The PI3K-Akt pathway is initiated by the activation of phosphoinositide 3-kinase (PI3K), which phosphorylates phosphatidylinositol 4,5-bisphosphate (PIP2) to generate phosphatidylinositol 3,4,5-trisphosphate (PIP3) [45]. PIP3 serves as a docking site for Akt (also known as protein kinase B), which is then phosphorylated and activated by phosphoinositide-dependent kinase 1 (PDK1) and mammalian target of rapamycin complex 2 (mTORC2) [46]. Once activated, Akt phosphorylates various downstream targets involved in cell survival, proliferation, and metabolism. In gastric cancer, the PI3K-Akt pathway is frequently dysregulated, leading to increased Akt activity and subsequent activation of downstream effectors [47]. Several studies have demonstrated the importance of the PI3K-Akt pathway in gastric cancer progression. Activation of Akt has been associated with increased cell proliferation, survival, and resistance to apoptosis in gastric cancer cells [48]. Additionally, activation of this pathway has been linked to enhanced invasion and metastasis of gastric cancer cells. Furthermore, aberrant activation of the PI3K-Akt pathway has been associated with resistance to chemotherapy and targeted therapies in gastric cancer. Inhibition of this pathway has been shown to sensitize gastric cancer cells to chemotherapy and enhance the efficacy of targeted therapies [49]. Notch and mTOR signaling pathways promote human gastric cancer cell proliferation. The study found enhanced expression of Notch pathway components, including Notch ligands, receptors, and downstream target genes, in human gastric adenocarcinomas [50]. Inhibition of Notch or mTOR signaling reduced the growth of human gastric cancer cell lines, indicating that both pathways are activated to promote gastric cancer cell proliferation. The study suggests a crosstalk between the ERα and Hh pathways in diffuse-type gastric cancer. Stimulation of the ERα pathway induced Sonic hedgehog (Shh) expression, activated the Hh pathway and promoted cell proliferation in ERα-positive gastric cancer cells [51]. These pathways play important roles in promoting cell growth and proliferation, and their dysregulation may contribute to gastric cancer development and progression. Further research is needed to fully understand these pathways’ intricate interactions and potential therapeutic implications in gastric cancer.

## 4. Materials and Methods

### 4.1. Compound Identification

TYTW created under the guidance of “tonifying yuan qi and detoxification” theory, consists of 9 herb species, including *R. pseudostellariae*, *O. japonicus*, *S. baicalensis*, *A. herba*, *C. aurantium*, *S. miltiorrhiza*, *A. valvata*, *C. lanceolatae*, and *G. preparate*. The nine herb species were screened to identify the composed ingredients and targets. Predictions of the compounds of herb species were investigated using ETCM (http://www.nrc.ac.cn:9090/ETCM/, accessed on 31 August 2023) [52], TCMSP (http://sm.nwsuaf.edu.cn/lsp/tcmsp.php, accessed on 31 August 2023) [53], BATMAN-TCM (http://bionet.ncpsb.org/batman-tcm/, accessed on 31 August 2023) [54], and SymMap (http://www.symmap.org/, accessed on 31 August 2023) [55]. Absorption, distribution, metabolism, and excretion (ADME) were employed as a computational evaluation model for pharmacokinetic research to filter drug-like compounds according to evaluation via the ETCM database.

### 4.2. Target Prediction and Collection for the Components

Canonical SMILES were acquired from the PubChem database (https://pubchem.ncbi.nlm.nih.gov/, accessed on 4 September 2023). Component targets were obtained either via searching the databases TCMSP, ETCM, BATMAN-TCM, and SymMap, or predicting through a search against TagetNet (http://targetnet.scbdd.com, accessed on 4 September 2023) [56], SwissTargetPrediction (http://www.swisstargetprediction.ch/, accessed on 4 September 2023) [57] and SEA (similarity ensemble approach, https://sea.bkslab.org/, accessed on 4 September 2023) with the target species setting as *Homo sapiens*, and duplications removed [58].

### 4.3. Identification of Thromboembolism-Related Targets

Dysregulated genes induced by gastric cancer were screened, selected, and obtained from the GeneCards, Malacards, and DisGeNET databases and other references. The targets related to gastric cancer were obtained from GeneCards and MalaCards, an integrated database of human maladies and their annotations, using “Gastric cancer” as the keyword. The disease targets were also acquired using “Gastric cancer” as keywords in the comprehensive DisGeNet gene–disease association database with integrated information from expert-curated repositories, GWAS catalogs, animal models, and the scientific literature. Finally, the intersections between genes retrieved from public databases (GeneCards, MalaCards, and DisGeNet) provided the disease targets associated with gastric cancer.

### 4.4. Construction of the PPI Network and HCTD Network

The following network and pathway analyses were performed on the overlapping targets between genes linked with gastric cancer and the target genes of the TYTW compounds. The overlapping targets were imported into the STRING platform (Version 11.0). The species was set to *Homo sapiens*, and the minimum required interaction score was set to the median confidence of 0.4 to retrieve concise PPI information for the subsequent analysis step. The HCTD network, built on interactions between TYTW compounds, gene symbols, and gastric cancer, was visualized using Cytoscape software (v3.10.1). The corresponding networks were analyzed using the Cytoscape plugin MCODE to acquire clusters of core PPI networks. Connected regions in PPI networks may represent a particular phenotype with unique biological functions. The various node shapes represent overlapping targets, compounds, herbs, and diseases. The nodes are linked by edges (lines), indicating interactions between nodes. As such, the topological parameters of a network can be used to predict the targets, while the candidate hub nodes analyzed by Cytoscape’s network analyzer tool can be identified by calculating the two topological features of betweenness and degree. Closeness centrality refers to the fraction of all shortest paths between nodes in a network, while degree indicates the number of connections. The top 6 nodes were collectively screened as the hub genes in the network with the criteria of closeness centrality > 0.5 and degree > 2.

### 4.5. GO and KEGG Analysis

The MCODE clustering was used for functional enrichment analysis of the targets of active compounds against gastric cancer. The enrichment analysis was conducted by analyzing GO and KEGG in Metascape. Metascape provides biologists with a resource for systematic dataset analysis as a web-based portal with comprehensive gene annotation and GO and KEGG pathway enrichment analysis. The pathway enrichment of putative proteins with a *p*-value > 0.01 was considered significant and of interest. The GO terms were sorted into biological process, molecular function, and cellular component to examine molecular activity, cellular role, and gene expression. Biological processes and pathways with the best *p*-values were colored based on the results obtained via Metascape. Enrichment analysis was also performed in DisGeNET to identify associated diseases. The results of the KEGG enrichment analysis were used to construct a KEGG pathway network to determine the proteins involved in the effects of TYTW. According to the STRING results, the gene pathway of TYTW against gastric cancer was constructed to delineate the biological pathways and key targets involved, to explore the potential mechanisms of TYTW in gastric cancer.

### 4.6. Receptor–Ligand Molecular Docking Simulations

Molecular docking is an efficient method to rapidly verify the correlation between active compounds and candidate target genes from the pharmacological network. Simulation mapping, a widely used technique in computer-aided drug design for hit identification, can show bioactive compounds that fit into target pockets and identify potential binding sites [59]. Receptor–ligand complexes of interest were examined by in silico molecular docking as follows. Firstly, the protein structure from the Protein Data Bank (PDB) was processed by removing solvents and organics in PyMol software (v2.5.5). Next, the compound or ligand’s structure data file (SDF) was acquired from PubChem and converted to PDB format. The compounds and the corresponding target proteins were subsequently docked in AutoDock Vina software (v1.1.2) to prepare PDBQT format files for target and ligand screening and grid and docking parameter files. According to the docking results, the degree of receptor–ligand binding can be judged by the binding affinity (energy level). The lower the energy, the greater the possibility of receptor–ligand interaction [60].

## 5. Conclusions

This study employed network pharmacology and molecular docking to investigate the mechanisms underlying the pharmacological activities of TYTW, traditional herbal medicine widely used in Asian countries like China, Japan, and Korea, and explore its therapeutic potential for treating gastric cancer. The results demonstrated that TYTW containing bioactive compounds, including agrimoniin, baicalin, corosolic acid, and luteolin, might mitigate gastric cancer via effects on the PI3K/AKT, gastric cancer, and MAPK signaling pathways and related biological processes. Furthermore, TYTW predominantly targeted AKT1, CASP3, ESR1, IL6, PIK3CA, and PTGS2, subsequently regulating the above pathways and biological processes. Therefore, this study represents preliminary research on the anticancer molecular mechanism of TYTW by performing network pharmacology and providing theoretical evidence for further experimental investigations. Given the inherent constraints of this study, which primarily depends on database predictions of drug actions derived through data mining, future validation via in vivo or in vitro studies is necessary.

## Figures and Tables

**Table 1:** ADEMT and drug-likeness of each metabolite of nine herb species based on ETCM.

Herb Species	Metabolites	Molecular Formula	Hepatotoxicity	Solubility	Absorption Level	Drug-Likeness Grading
*Radix pseudostellariae*	1-Monolinolein	C_2_0H_36_O_4_	0	−3.058	0	Weak
	Acacetin	C_16_H_12_O_5_	1	−3.413	0	Good
	Beta-sitosterol	C_29_H_50_O	1	−8.256	3	Weak
	Luteolin	C_15_H_10_O_6_	1	−2.856	0	Moderate
	Schottenol	C_29_H_50_O	* N/A	N/A	N/A	N/A
	Taraxerol	C_30_H_50_O	1	−8.8	3	Weak
*Ophiopogon japonicus*	Ruscogenin	C_27_H_42_O_4_	1	−5.062	0	Moderate
	Ophiopogonanone B	C_18_H_18_O_5_	1	−4.053	0	Good
	Linalool	C_10_H_18_O	0	−2.194	0	Moderate
	Adenosine	C_10_H_13_N_5_O_4_	1	−0.316	2	Moderate
	Wogonin	C_16_H_12_O_5_	1	−3.428	0	Good
*Scutellaria baicalensis*	Tetramethoxyflavone	C_19_H_18_O_6_	1	−4.326	0	Good
	Scutevulin	C_16_H_12_O_6_	1	−3.207	0	Good
	Dimethoxyflavone	C_17_H_14_O_7_	1	−3.414	0	Good
	Baicalin	C_21_H_18_O_11_	1	−3.506	3	Weak
	Baicalein	C_15_H_10_O_5_	1	−2.976	0	Good
	7-Methoxybaicalein	C_16_H_12_O_5_	1	−3.415	0	Good
*Agrimoniae herba*	Agrimoniin	C_82_H_54_O_52_	N/A	N/A	N/A	N/A
	Catechin	C_15_H_14_O_6_	1	−2.445	0	Moderate
	Dihydroquercetin	C_15_H_12_O_7_	1	−2.492	1	Moderate
	Quercetin	C_15_H_10_O_7_	1	−2.633	1	Moderate
*Citrus aurantium*	Prunasin	C_14_H_17_NO_6_	0	−0.093	1	Moderate
	Neohesperidin	C_28_H_34_O_15_	1	−4.715	3	Weak
	Naringin	C_27_H_32_O_14_	1	−4.385	3	Weak
	Hesperetin	C_16_H_14_O_6_	1	−3.157	0	Good
	Ephedrine	C_10_H_15_NO	0	−1.169	0	Good
*Salvia miltiorrhiza*	Cryptotanshinone	C_19_H_20_O_3_	0	−5.673	0	Good
	Danshenol A	C_21_H_20_O_4_	1	−4.226	0	Good
	Danshenol B	C_22_H_26_O_4_	0	−4.619	0	Good
	Danshensu	C_9_H_10_O_5_	1	−0.345	0	Moderate
	Isocryptotanshinone	C_19_H_20_O_3_	0	−5.68	0	Good
	Isotanshinone I	C_18_H_12_O_3_	1	−6.049	0	Moderate
	Salviol	C_20_H_30_O_2_	0	−5.101	0	Good
	Tanshinol A	C_18_H_14_O_4_	1	−3.556	0	Good
	Tanshinone I	C_18_H_12_O_3_	1	−6.05	0	Moderate
*Actinidia valvata*	Asiatic acid	C_30_H_48_O_5_	1	−5.298	1	Weak
	Corosolic acid	C_30_H_48_O_4_	N/A	N/A	N/A	N/A
	Netursen-28-oic acid	C_30_H_48_O_5_	N/A	N/A	N/A	N/A
*Radix codonopsis lanceolatae*	Cycloartenol	C_30_H_50_O	0	−8.421	3	Weak
	Echinocystic acid	C_30_H_48_O_4_	1	−6.356	1	Weak
	Oleanolic acid	C_30_H_48_O_3_	1	−7.612	1	Weak
*Radix glycyrrhizae preparata*	Glycyrrhizic acid	C_42_H_62_O_16_	1	−6.703	3	Weak
	Isoliquiritin	C_21_H_22_O_9_	1	−2.288	3	Weak
	Liquiritigenin	C_15_H_12_O_4_	1	−3.234	0	Good
	Liquiritin	C_21_H_22_O_9_	1	−2.432	2	Weak

* N/A represents not available.

**Table 2:** Selection of the most significantly enriched and specific GO and KEGG terms in the network modules.

Module	GO Terms/KEGG Terms	LogP	Total Genes	Mapped Genes
1	Pathways in cancer	−48.7	531	35
	CKAP4 signaling pathway map	−40.5	116	24
	Lipid and atherosclerosis	−44.1	215	27
	Malignant pleural mesothelioma	−35.0	440	27
	PI3K-Akt signaling pathway	−32.2	354	24
	Endocrine resistance	−31.9	18	98
	Response to hormone	−31.8	29	788
	Proteoglycans in cancer	−31.8	21	205
	Hepatitis C and hepatocellular carcinoma	−31.7	16	56
	Photodynamic therapy-induced NF-kB survival signaling	−30.1	35	14
2	Regulation of mitotic cell cycle phase transition	−7.9	355	5
	Regulation of cell cycle phase transition	−7.4	456	5
	Pathways in cancer	−7.05	531	5
	Cellular response to organic cyclic compound	−5.3	500	4
	Nucleic acid-templated transcription	−3.4	594	3
3	Nuclear division	−7.9	308	4
	Meiotic cell cycle process	−6.0	182	3
	Sister chromatid segregation	−6.0	140	3
4	Cellular responses to stress	−4.7	788	4

**Table 3:** Selection of the most significantly enriched GO and KEGG terms in functional clustering in DAVID.

Cluster	Term	Count	*p*-Value
Disease enrichment	Stomach neoplasms	29	7.15 × $10^{-19}$
	Malignant neoplasm of stomach	29	9.36 × $10^{-19}$
	Hereditary diffuse gastric cancer	29	6.02 × $10^{-18}$
KEGG pathway	PI3K-Akt signaling pathway	33	7.33 × $10^{-20}$
	Kaposi sarcoma-associated herpesvirus infection	25	4.67 × $10^{-18}$
	Small cell lung cancer	17	4.72 × $10^{-14}$
	Proteoglycans in cancer	25	1.41 × $10^{-17}$
	MAPK signaling pathway	26	1.41 × $10^{-14}$
	Thyroid-stimulating hormone (TSH) signaling pathway	13	2.87 × $10^{-11}$
G.O. terms	Protein phosp+B51:B123horylation	29	1.05 × $10^{-19}$
	Protein serine/threonine/tyrosine kinase activity	27	2.26 × $10^{-19}$
	ATP binding	39	2.83 × $10^{-15}$
	Protein serine/threonine kinase activity	21	1.13 × $10^{-13}$
	Peptidyl-serine phosphorylation	15	2.26 × $10^{-12}$
	Protein kinase activity	19	5.35 × $10^{-12}$
	Kinase activity	13	8.14 × $10^{-9}$

**Table 4:** Summary of binding affinity of each component.

Species	Components	Targets	Binding Affinity (kcal/mol)
*Agrimoniae herba*	Agrimoniin	AKT1	−11.3
		CASP3	−8.9
		ESR1	−9.6
		IL6	−8.3
		PIK3CA	−12.0
		PTGS2	−10.9
*Scutellaria baicalensis*	Baicalin	AKT1	−8.6
		CASP3	−7.8
		ESR1	−7.3
		IL6	−6.7
		PIK3CA	−9.0
		PTGS2	−10.0
*Actinidia valvata*	Corosolic acid	AKT1	−8.2
		CASP3	−8.1
		ESR1	−7.6
		IL6	−6.8
		PIK3CA	−9.9
		PTGS2	−8.3
*Radix pseudostellariae*	Luteolin	AKT1	−8.2
		CASP3	−6.9
		ESR1	−7.5
		IL6	−7.0
		PIK3CA	−9.3
		PTGS2	−9.6

## Data Availability

Data are contained within this article.
